# Correspondence

**Published:** 1883-08

**Authors:** 


### Correspondence.

*Editor Register:*

There recently appeared in a newspaper of Atlanta an item which might well be considered by our profession, though it did not directly pertain to medicine.

Suit was brought by a railroad man against a merchant and his attorney for damages sustained by him in a garnishment of his wages. This garnishment, as I understood it, was an attempt to force payment for provisions sold the railroader. He was doubtless insulted that he should be expected to pay his debts, and may have been very slightly damaged by the holding up for some days of his wages; but it does seem hard that the merchant, already injured by his dishonest customer, should be further injured, and, as I conceive, insulted by the court, in this granting of heavy damages (several hundred dollars) for the very natnral desire to obtain his rightful money. I cannot say the decision was not legal; but I firmly believe it was not just.

Garnishment has frequently and successfully been practiced in our city for obtaining medical fees; and the knowledge that it might be practiced has often enabled the physician to enforce payment of his just claim. But this decision probably deals a death-blow to this method of collection, and still further enlarges the already very large loop-hole of escape that the law provides for dishonest debtors.

Possibly some hardship might be worked by the execution of garnishment; but I cannot think anyone should be encouraged in making debts that he will not, or even cannot, pay. I must think it time the profession was endowed with some rights that others are bound to respect I have never found that doctors are disposed to unkindly push a patient who evinces even a desire to be reasonably honest.

Now for the deduction from the above lengthy text: It is time that we were improving our methods of dealing with a certain class of patrons, for the law will not afford us any protection. We are too easily taken in by every passing stranger. I know that the boldest impositions are constantly effected by an irresponsible class, even in office practice. There may be some reason for a degree of submission to it in a general practice; but in office work it is totally inexcusable. Such patients are allowed to make the round of physicians, and escape payment in almost every instance. Pay is often not demanded, and the impostor stalks out, sometimes not even deigning to leave his address; or at the best, soothing his expectant victim with the gentle lie that he "will call again and make it all right."

I believe that in our overgrown village this imposition is more readily submitted to than in larger and older cities. In these, office work is regarded as cash work, and the pay is generally demanded, unless the patient's high credit is well established. The idea of entering a charge on the books against a transient or a but little known patient, is rightly regarded as preposterous.

The object of this paper is to encourage in our city the custom of collecting cash fees for all office work, unless, as before said, the patient be a regular and prompt-paying patron. Let us cease to be easy dupes of such systematic swindlers. We not only lose our money, but often are ridiculed with a chuckle of delight for being so softly taken in by the transparent trick. A politeness so refined as to forbid a demand for the fee under these circumstances is almost as delicate as that of the man who hesitated to stop another from kissing his (the man's) wife, because he had not been introduced to him.

It would seem well that an uniform rule be established among us by some concert of action. It might be advisable for each one to announce such rule by a placard posted in his office. The terms should be understood before the treatment of the case is undertaken. If they are not satisfactory we may lose a patient, but we would lose no money that otherwise would be gained. Moreover, we would avoid the cumbering of our books with accounts that are worthless, and the worrying with patients whose practice is undesirable.

Respectfully, JNO. THAD. JOHNSON.

---

*To the President and Members of the Medical Association of the State of Georgia:*

That this organization is beneficial to the medical profession, and consequently to the public, no thoughtful man can doubt. All proper measures adding to its membership extends its benefits. Now, what does its history teach us? Certainly this: In its early days the door was opened wide and terms of membership made easy. Consequently the number increased largely; from town and country, rich and poor, writers and speakers, listeners and learners. But a change came. Generous men, of ample means and paying practice, unacquainted with the war-crippled condition of their country brethren, but with a laudable ambition to appear well before the public in their Transactions, increased the annual dues to $5. Now, if I am correctly informed, this lessened the membership several hundred. To repair this damage the fee was reduced to $1 for all who could not come, and $2 for such as enjoyed the double benefits of attending the sessions and receiving the Transactions. Under this change prosperity was gradually returning; when, presto! the fee is again increased, and the worthy country doctor, of large practice and small pay, lingers and hesitates between the mortifying alternative of forfeiting membership or applying the fee to the necessities of his family. These vibrations between $1 and $5 do harm. There are other troubles enough. For even skill, eminence, reputation and large practice interpose difficulties. But these can be obviated by the city doctor, who may call in his neighbor, equally eminent and acceptable, to take charge of his patients for a few days. Not so with his country brother, who has no proxy at hand, and would deserve the censure he would be sure to receive if he were to leave without a suitable substitute. Another point: railroad facilities are not within easy reach of many of our country brethren; and again: before the war negro practice was certain pay. But now few negroes pay anything. And worse still, their former owners—at least many of them—pay little, if anything. The subject is fruitful, but I forbear, as I only wish to solve the problem, and bring back prosperity by the following means, namely:

1. Restore the $1 and $2 rule, lately repealed.

2. Publish the Transactions in cheap, light form, easily distributed through the mails; not in heavy, showy, costly binding, as if intended for a parlor ornament.

3. Continue the usual annual notices, for dues, to delinquents, but without the mortifying, humiliating threat of being deposed from membership in default of payment.

4. I will be one of fifty (or any other number the Association may designate) to pay $5 per annum, as dues, for life—or longer if needed.

5. When, from death, resignation, or otherwise, vacancies occur, let volunteers be called for, at the next annual session, to fill such vacancies.

Respectfully submitted,

HENRY GAITHER, M.D.

Oxford, Ga., July, 1883.

